# Assessing Attachment Representations in Adolescents: Discriminant Validation of the Adult Attachment Projective Picture System

**DOI:** 10.1007/s10578-016-0639-2

**Published:** 2016-03-26

**Authors:** Manuela Gander, Carol George, Dan Pokorny, Anna Buchheim

**Affiliations:** 1 Institute of Psychology, University of Innsbruck, 6020 Innsbruck, Austria; 2 Psychology Department, Mills College, Oakland, CA 94613 USA; 3 Psychosomatic Medicine and Psychotherapy, Ulm University, 89081 Ulm, Germany; 4 Department of Child and Adolescent Psychiatry, Medical University of Innsbruck, 6020 Innsbruck, Austria

**Keywords:** Adolescence, Attachment representation, Adult Attachment Projective Projective Picture System, Clinical attachment research, Validity

## Abstract

The contribution of attachment to human development and clinical risk is well established for children and adults, yet there is relatively limited knowledge about attachment in adolescence due to the poor availability of construct valid measures. The Adult Attachment Projective Picture System (AAP) is a reliable and valid instrument to assess adult attachment status. This study examines for the first time the discriminant validity of the AAP in adolescents. In our sample of 79 teenagers between 15 and 18 years, 42 % were classified as secure, 34 % as insecure-dismissing, 13 % as insecure-preoccupied and 11 % as unresolved. The results demonstrated discriminant validity for using the AAP in that age group, with no associations between attachment classifications and verbal intelligence, social desirability, story length or sociodemographic variables. These results poise the AAP to be used in clinical intervention and large-scale research investigating normative and atypical developmental correlates and sequelae of attachment, including psychopathology in adolescence.

## Introduction

The study of attachment, its assessment and clinical applications during adolescence promises to provide a far-reaching insight into underlying mechanisms of personality development and early psychopathology [1–4]. Adolescence is a period of profound transformation during which a major goal is to develop an integrated sense of self and autonomy from parents [5]. This process balances establishing culturally defined parental distance while maintaining trust in parents’ availability, responsiveness, and sensitivity. Autonomy is successfully reached through open communication of emotional states and thoughts of each member of the child-parent dyad [6]. The quality of parent–child attachment relationships in adolescence is fundamental to well-being. Longitudinal attachment studies have shown that childhood attachment security is associated with positive interaction in relationships in early adolescence (parents, friendships, romantic partners) and reduced likelihood of developing problematic behavior [6]. Childhood attachment security and insecurity appear to be buffer and risk factors respectively for cognitive processes, relationship interactions, conflict management, emotional regulation, depression, suicidal behavior, and residential and inpatient treatment [6–8].

Even though attachment theory provides a useful framework for understanding developmental processes and transitions, there is a paucity of research on adolescents [9]. To date, research has focused historically on children and adults as there are relatively few validated assessment options for adolescents. Although an increasing number of research papers have addressed the relevance of attachment issues for adolescence, the measurement gap makes it difficult to examine possible relationships between adolescent attachment and psychopathology [4, 7, 10, 11]. The very few published studies indicate that the unresolved attachment pattern- a category that can only be assessed using a narrative attachment measure-might play a key role for the onset of mental disorders in adolescence [2, 8].

Problems in establishing well-validated assessment measures for adolescents have limited ability examining attachment patterns and correlates in this age group. As compared with well-established measures for children [1], there are relatively few attachment measures for adolescents that are designed following Bowlby’s [12] tenants for attachment assessment. He proposed that assessment must address who attachment figures are, their accessibility, and an individual’s confidence that attachment figures will provide protection, support, and comfort. More recently, attachment experts have suggested that assessment for adolescents should also include evaluation of their potential for collaborative and balanced negotiations, which are related to caregiving sensitivity and partnership flexibility [13]. Even though observational measures of attachment behavior are frequently used in childhood, behavioral assessments of adolescent attachment are underrepresented in the literature. To date, the Goal-Corrected Partnership in Adolescence Coding System (GPACS)-designed to assess forms of disorganized attachment behaviors during parent-adolescent interaction tasks-remains one of the only available behavioral assessments for this age group [14, 15] In the current literature, two kinds of adolescent assessments predominate in the field: self-report and interview. There are numerous self-report questionnaires of attachment for adolescents [1]. These measures vary in conceptual validity and range in operational definitions and the dimensions used to define attachment security. One set of self-report measures assesses romantic “attachment style,” a model derived from personality theory applied to romantic relationships that has no demonstrated empirical link to developmental attachment [16]. Another set of questionnaires was designed to assess parent–child attachment by asking questions about perceived attachment to caregivers. There are a number of well-validated questionnaires like the Attachment Security Scale [17, 18], the Inventory or Parent and Peer Attachment-Revised [19], the adolescent version of the Experiences in Close Relationships Scale-Revised [20] or the Kenny’ Parental Attachment Questionnaire [21] for that age group. While these instruments are administrator-friendly (e.g., they require no training for administration and scoring) and demonstrate acceptable standards for empirical validity, the lack of established alignment with developmental attachment assessments for children and adults introduces confusion for interpretation and integration in developmental theory and clinical application [1]. The other form of measurement is representational interviews that allow us to look beyond conscious thoughts of relationships by analyzing the mental organization of discourse when people talk about attachment experiences. The most popular is the Adult Attachment Interview (AAI) [22, 23]. Described in the field of attachment as the “gold standard” for attachment assessment, the AAI has been used with adolescents for almost three decades. The primary goal of the AAI and its “downward” versions—the Attachment Interview for Childhood and Adolescence [24] and the adolescent form of the Child Attachment Interview (CAI) [25]—is to establish consistency with the developmental classification nosology for adults. The CAI is a revised version of the AAI with minor adaptions like a simplified language, and a removal of the questions dealing with parents’ relationship to their offspring [24]. Based on verbatim transcripts, adolescents can be classified as secure-integrated, dismissing, preoccupied, and unresolved (disorganized in children). In addition to the AAI, there is an adolescent version of the Attachment Script Assessment (ASA), a prompt-word outline method to assess the degree of security when producing narratives [26, 27]. Even though these narrative instruments have demonstrated acceptable standards for validity in adolescence, some researchers have expressed concerns on different issues [25, 28]. First, similar to self-report measures, individuals probably “edit” their narrative to fit the occasion, which may be especially a problem with adolescents [25, 29]. Furthermore, the CAI has demonstrated an underrepresentation of the preoccupied attachment pattern in young people due to their use of extensive examples, coherent descriptions and emotional openness that might easily be miscoded as a secure attachment pattern [30]. From a practical standpoint, the AAI and CAI are also not quite easily applicable especially in the clinical context as the interview procedure and transcription required for analysis are time consuming and costly [31]. There have also been recent concerns that the AAI was originally developed to measure adult’s caregiving capacity and ability to raise secure infants that might not be equivalent to being secure with one’s own attachment figures in adolescence [32].

A viable alternative to self-report and interview adolescent assessments is the Adult Attachment Projective Picture System (AAP) [31, 33]. The AAP provides attachment classifications based on the analysis of “story” responses to a set of theoretically-derived attachment-related drawings of scenes. All scenes depict theoretically defined attachment situations, such as solitude, illness, separation, death, and potential maltreatment. Story analysis includes evaluation of all of the attachment dimensions described earlier. The AAP has several advantages over other measures: (1) it circumvents the problem of potential “editing” because it never asks an individual to describe his or her own real life experience; (2) it is economical and user friendly in administration and coding; and (3) it is feasible for experimental settings and is one of the only attachment measurements activating the attachment system that has repeatedly and successfully been used in an fMRI setting [34]. Furthermore, the AAP enables researchers and clinicians to examine new construct-based features of attachment derived from Bowlby’s theoretical discussions that cannot be assessed using other narrative instruments like the AAI. These include (1) the assessment of defensive processing which provides valuable state of mind information not only for the purpose of classification but also for clinical application [34, 35], (2) a qualitative analysis of attachment-related traumatic material underlying the unresolved attachment status [34, 35], (3) the dimensional coding of agency of the self [36, 37], that is the degree of conscious evaluation and reorganization of attachment-related experiences that Bowlby [38] considered as essential for mental health, and (4) measuring differences in response to monadic stimuli representing aloneness and dyadic stimuli depicting interactions in attachment relationships [34, 35]. These new construct-based coding dimensions of the AAP have already demonstrated clinical relevance and interesting findings in adult samples [39]. Using this instrument in adolescence might provide a unique insight into attachment-related developmental issues and its relationship to psychopathology in that age group.

To date, only very limited data on the discriminant validity of narrative measures developed for assessing attachment in adolescents is currently available [40]. Most attachment studies therefore use self-report measures, whereas studies on attachment in adolescents employing narrative techniques like the AAI are often limited as the interview procedure and the coding are very time-consuming. The AAP might circumvent these practical drawbacks as this instrument requires less time for administration and coding but at the same time shows an impressive agreement to the AAI [41]. An initial step to assess attachment representations in adolescents with the AAP is to validate its use. Assessing discriminant validity is one of the most important issues when testing psychometric properties of narrative measures as they evaluate individual differences in the discourse characteristics, which may be related to dimensions of intelligence, social desirability and sociodemographic characteristics of participants.

## Aim of the Study

The purpose of the present study is to examine the discriminant validity of the AAP in adolescence. To establish the AAP as an assessment for adolescents, we first analyze the distribution of classification groups in relation to those established for adolescents using the AAI [28]. A distribution that is remarkably different from those reported in AAI studies, would question our sample and our findings on validity from the very start of the study. In a second step, we investigate possible relations between attachment patterns, verbal intelligence and fluency. Especially in adolescence, verbal intelligence and other cognitive variables can provide plausible alternative interpretations or they can represent important covariates when narrative attachment instruments are used [42]. Examining associations with verbal intelligence is uniquely important for adolescents as studies have demonstrated that, although IQ seems to be stable across the lifespan, verbal IQ can fluctuate in teenage years due to variations in the brain structure [43]. Regarding verbal intelligence, it is possible that adolescents with high verbal intelligence (i.e., good vocabulary, synonyms, and sophisticated word use) could be judged secure because they show fewer logical inconsistencies in their stories and do not evidence discourse elements usually associated with insecurity (e.g., confusion, contradiction, story line shifts, truncated short responses) turning their narratives into more elaborated and thus longer storylines. These elements can also lengthen the narrative that is produced, introducing a potential confound between story length and attachment pattern. Indeed, for some instruments, individuals who tell longer and richer stories receive higher scores than storytellers who give shorter and more descriptive answers. However, the length of the AAP stories might also be a confounding variable for the insecure-preoccupied attachment. These individuals are unable to integrate opposing representations of the self and attachment figures, their AAP responses are often laden with unnecessary details, contradictory elements and multiple storylines that can also lengthen the narrative that is produced. We therefore examined the participant’s verbal fluency in relation to attachment classification, operationalized as the story length expressed by the logarithm (see below) of the number of words in the transcribed AAP protocol.

A third important challenge for the validity of the AAP is the possible relationship between social desirability and attachment classifications. Narrative techniques bear the risk of assessing social adjustment rather than a defined construct. Adolescents are known to be particularly concerned with acceptance by adults and peers and fulfilling their need for belonging by presenting themselves in a social desirable light [44]. As the AAP picture stimuli are designed to elicit distress (i.e., activate attachment), adolescents may shape their responses to pictures of distressing situations in socially desirable ways. This potential confound would be especially problematic for the dismissing attachment pattern, which is defined by minimizing attachment distress and the need for attachment figures [23]. Indeed, the social desirability problem has already been suggested for dismissing adolescents when using the AAI [42, 45]. In the AAP, dismissing participants tell stories that often demonstrate themes of turning away from attachment figures; they evaluate themselves as strong and unaffected by life’s stressors, attempting to divert or neutralize affective reactions that are triggered by the threatening events (e.g., death or illness) depicted on the AAP pictures [31]. Furthermore, studies have demonstrated that adolescents with good language skills tend to behave in socially desirable ways, such as showing less oppositional, non-compliant and aggressive behavior than adolescents with poorer language skills [46]. Therefore, this study will also test a possible interaction between social desirability and verbal intelligence in our adolescent sample. As social desirability and verbal intelligence might be intertwined with sociodemographic factors like educational level, gender or age we additionally tested possible relations of these variables to attachment groups.

Lastly, we were interested if attachment patterns are associated with sociodemographic variables like gender, socioeconomic background and age. It is a widely held belief in the literature, that especially adolescent males exhibit a more dismissive stance towards attachment experiences. In a recently published meta-analysis Del Giudice [47] outlined that studies on primarily romantic attachment using self-report questionnaires indeed found differences, especially among young males [48, 49]. It was speculated that gender-specific reproductive strategies might cause these observed differences. However, the majority of AAI studies demonstrated that attachment classifications measured with a narrative instrument are largely invariant across gender [28, 50] suggesting that the sexual component in intimate relationships might be more affected by gender than mental representations of the past experiences with caregivers [50]. Nevertheless, gender issues represent an important challenge for validity of the AAP in adolescence as they seem to be quite apparent in this age group when using questionnaires of attachment but not when using narrative instruments. A further issue challenging the validity addresses possible associations between attachment patterns and socioeconomic background. A considerable number of studies have reported the universality of secure attachment patterns among different backgrounds [28] as attachment security depends on experiences of sensitivity and responsiveness of attachment figures and not on living conditions, parental family status, amount of siblings or level of education. Finally, the literature suggests that developmental transformations might cause shifts from one insecure attachment classification to another. However, attachment security is supposed to be stable across age. Even though we do know that the dismissing attachment pattern is slightly overrepresented in adolescent samples due to autonomy strivings [50], the relationships between most adolescents and their parents do not seem to change drastically [11]. Based on studies that found significant stability of secure attachment representations from infancy to adulthood [11], we suppose that age is not related to attachment groups.

In sum we hypothesize that (1) the distribution of attachment classification groups is analogues to those reported in AAI studies of non-clinical adolescents, (2) that attachment classifications are not associated with verbal intelligence and fluency (3) that there are no relations between attachment groups, social desirability and sociodemographic variables (gender, household, marital status of parents, educational level, age and amount of siblings).

## Method

### Participants

The initial sample was comprised of 95 adolescents ranging in age between 15 and 18 years by the last birthday. Participants lived in communities in different areas of Austria and southern Germany. The sample was recruited using flyers and email. A total of 84 met the inclusion criteria (a sufficient knowledge of the German language and an appropriate literacy to fill out the questionnaires). The study procedures were approved by the institutional review board. We obtained IRB approved informed consent from the adolescent’s parents as well as the adolescent’s assent. Five participants were excluded due to incomplete questionnaires.

The final sample consisted of 79 adolescent participants (58 girls and 21 boys) with a mean age of 16.78 years (*SD* = 1.03). Ninety one percent of the participants were aspiring for or had passed the examination for a higher education degree (“Matura”). The majority of participants attended school (80 %), however some adolescents did not attend school because of full- and part-time employment. Most of the participants came from families with parents who were married or partnered (90 %) and lived in their family home (85 %), and had siblings (90 %; mean = 1.57, *SD* = 0.94). Those not living at home lived in apartments with friends, siblings or alone.

### Measures

#### Adult Attachment Projective Picture System

The AAP [31] assesses adult attachment status using a set of picture stimuli. The stimulus set includes eight line drawings, a warm-up picture and seven attachment scenes of individuals in attachment situations when they are alone or in potential attachment dyads. Participants are asked to tell a story regarding the depicted characters in each scene, guided by a series of standardized prompts that ask: what is happening in the scene, what led up to the scene, what the characters are thinking or feeling, and what happens in the end? The AAP interviews are audio-recorded and analyses are done from verbatim transcripts.

Each stimulus response is coded for content and defense. The alone picture responses (i.e., stimuli that portray individuals alone) are evaluated for *agency of self* and *connectedness. Agency of self* evaluates the capacity for attachment relationships to foster productive action. These are coded for three levels ranging from integrated to functional to absence. *Connectedness* evaluates the representational desire to be in relationships with others. The dyadic picture responses (i.e., stimuli that portray individuals in attachment dyads) are evaluated for *synchrony. Synchrony* assesses balance and mutuality in attachment-caregiving relationships. The AAP additionally evaluates the three forms of defense (following Bowlby [38]) for each picture stimuli: *deactivation*, *cognitive* *disconnection*, and *segregated* *systems*. *Deactivation* describes story actions or evaluations that shift attention away from attachment distress, thus attempting to eliminate the need to address attachment relationships and distress as important. *Cognitive disconnection,* in contrast to deactivation, describes story actions that manage distress by attempting to separate attachment-related emotion from events and the people that arouse emotion. This defense works to create a representational smoke screen for distress, which is associated with being preoccupied with and entangled in relationships and craving for intimacy. S*egregated systems* describe evidence in the response of being frightened and threats to self. According to attachment theory, segregated systems defenses attempt to block frightening attachment experiences and affect from the consciousness because they threaten integrity and risk dysregulation of self [38, 51]. Finally, responses are evaluated for personal experience, which evidences blurring of self-other boundaries by leaking descriptions of personal experience while telling hypothetical stories. This evaluation is especially important to determine evidence of lingering traumatic attachment dysregulation that appears in the autobiographical experience (for more information on these scales see George and West [31, 39]).

Attachment groups are designated by evaluating the overall pattern of content and defense coding for the attachment stimuli. Secure attachment (F) is characterized by evidence of integrated agency, connectedness, and synchrony. Insecure dismissing (Ds) and preoccupied attachment (E) are characterized by the predominance of agency of self, connectedness, and synchrony that is functional or absent with a prevalence of either deactivating defenses (as indicated by focus on themes of achievement, personal strength, neutralization, problem solving, or rejection in the AAP responses) or disconnecting defenses (as indicated by themes that include withdrawal, anger, uncertainty and confusion in the AAP responses) respectively. Insecure-unresolved attachment (U) is characterized by the failure to re-organize contain (i.e., regulate) evidence of segregated systems in a response. The coding can only be done by a certified AAP rater who completed a 9-day intensive workshop on the AAP coding system and classification procedure and required 80 % concurrence with a minimum of 30 standard reliability cases.

Studies provide evidence of excellent concurrent validity of the AAP with the AAI, test–retest reliability, interjudge reliability and discriminant validity in healthy controls and clinical patients. Results from a large-scale psychometric investigation including 144 adult participants demonstrate excellent inter-judge reliability; the concordance rate for two judges on the four-group classifications were 90 %, κ = .85, *p* < .001, test–retest reliability (after three months 84 % remained in the same attachment category; κ = .78, *p* < .001). To evaluate the concurrent validity, AAP classifications were compared to independent AAI classifications. The concordance rates for the four-group classifications were 90 %, κ = .84, *p* < .001 and for the two groups (secure vs. insecure) even 97 %, κ = .89, *p* < .001 [31, 33, 52]. Furthermore, the AAP demonstrated a satisfying discriminant validity in adults. For additional psychometric data independent from collaborative work with AAP developers see for example Benoit, Bouthillier, Moss, Rosseau, and Brunet [36] and Beliveau and Moss [53].

#### Hamburg Wechsler Intelligence Scale

The Hamburg Wechsler Intelligence Scale for Children (HAWIK-IV) [54] and German version of the Wechsler Adult Intelligence Scale III (HAWIE-R) [55] were used in the present study to assess verbal intelligence in participants younger and older than 16.11 years, respectively. These two intelligence scales are the German modifications of the WISC [56] and WAIS [57]. The HAWIK-IV verbal comprehension index (VCI) is derived from subtests that measure verbal reasoning and comprehension. The HAWIE-R includes six verbal and five performance subtests. The results of the verbal IQ from the HAWIE-R are comparable to the VCI of the HAWIK-IV [54]. The HAWIK-IV shows acceptable internal consistency (α = .88 for processing speed to α = .97 for the full scale). Results from test–retest reliability demonstrate that the mean retest scores for all subtests are higher than the mean test scores from the first administration with effect sizes ranging from .08 (comprehension) to .60 (picture completion). Correlations between the HAWIK-IV verbal comprehension index and its predecessor WISC-III verbal IQ are *r* = .87 and *r* = .74. Furthermore, the HAWIK-IV demonstrated an acceptable relation to measures of achievement, memory, adaptive behavior, emotional intelligence, and giftedness in children and adolescents [56, 58, 59]. The HAWIE-R also demonstrated satisfying internal consistency (α = .95 to .97) and test–retest reliability between *r* = .70 (7 subscales) to *r* = .90 (2 subscales). Furthermore, it correlated highly with the Stanford-Binet IV test (*r* = .88) and had established acceptable concurrent validity with other achievement measures of memory, attention, cognitive ability and language [55, 57].

#### Balanced Inventory of Desirable Responding

The BIDR [60, 61] is a two-factor inventory that assesses in a 20-item questionnaire using a 7-point Likert scale (1 = not true to 7 = very true) social desirable responding, designed to reflect an individual’s tendency to deny socially undesirable traits and to portray the speaker in a favorable light. The BIDR measures two components of social desirability: self-deceptive enhancement and impression management. Psychometric properties demonstrate a high test–retest reliability (self-deception *r* = .69; impression management *r* = .65) and a satisfactory internal consistency (self-deception range α = .68 to .80; impression management α = .68 to .86; social desirability α = .76 to .84) [61]. In the study by Musch et al. [60], the German version of the BIDR also demonstrated satisfactory psychometric qualities. Internal consistency was demonstrated to be satisfactory (α ranging from .64 to .66). In our sample, the internal consistency of the BIDR was acceptable (Cronbach’s alpha α = .63). In German reliability studies, the self-deceptive enhancement scale of the BIDR showed significant convergent validity with the lie scale of the Eysenck Personality Inventory (r_s_ = .43, *p* < .001) and the social desirability scale of Mummendey and Eifler [62] (r_s_ = .41, *p* < .001 for the self-deceptive enhancement scale). Furthermore, there was no significant correlation between the BIDR scores and subjectively reported school grades [60].

### Procedure

Participants and their parents gave their informed consent after receiving a complete verbal and written description of the study and assurance of anonymity, with assessments only accessible to members of the research team. Participants were scheduled for a single testing session at the research laboratory located at the Institute of Psychology at the University of Innsbruck. Here they completed the study measures and a sociodemographic questionnaire in the order described below. All tests were administered in a comfortable and quiet room. Participants received 30 € for completing the study.

The AAP was administered by one of three psychology students trained in administration technique by a certified AAP judge (six practice training cases under supervision). For this study, two certified reliable AAP judges (MG, AB) independently coded all AAP transcripts; one of them was unaware of the data and hypotheses. The inter-rater reliability analysis demonstrated empirically a very high concordance; the kappa for the four-group classification was κ = .96 with a narrow 95 %-confidence interval [0.91, 1.00], *p* < .001. The high concordance was found also for all particular attachment classes F secure (κ = .95, [0.88, 1.00]), Ds dismissing (κ = .94, [0.87, 1.00]), E preoccupied (κ = 1.00), and U unresolved trauma (κ = 1.00). In fact, the both independent raters agreed in as many as 77 out of N = 79 cases of this study.

A statistical power analysis was performed to check the sufficiency of the selected sample size for the particularly investigated comparison of secure and insecure attachment groups. We considered significance as α = 0.05 and the power as 1 − β = 0.80. There were two identified groups, their sample sizes were n_1_ = 33 (secure) and n_2_ = 46 (insecure). The power analysis has shown that—by the given group sample sizes—group differences in score values between both groups would be likely found significant when expected effect size exceeded the value of the Cohen’s d ≥ 0.58, corresponding to η^2^ = 0.076. The study focussed on the discriminant validity; the aim was to show that the associations between AAP classification and psychometrical scales are considerably low. Both for the 2-groups and 4-group AAP classification, we demonstrated this by confidence intervals for the η^2^ coefficient.

## Results

### Attachment Representation Distribution

The attachment classification distribution in our sample of adolescents was as follows: 42 % secure, 34 % insecure-dismissing, 13 % insecure-preoccupied, and 11 % insecure-unresolved. Gender distributions showed that 47 % of the boys and 40 % of the girls were classified secure, 29 % of the boys and 36 % of the girls were classified dismissing, 10 % of the boys and 14 % of the girls were classified preoccupied; and 14 % of the boys and 10 % of the girls were unresolved. This distribution is analogous to the distribution reported in a meta-analysis of adolescent attachment classifications in community samples using the AAI: 44 % secure, 34 % dismissing, 11 % preoccupied, and 11 % unresolved [28].

### Sociodemographic Descriptive Variables

Prior to hypothesis testing, we examined the differences in 2-group and 4-group attachment classifications using six sociodemographic variables (gender, household, marital status of parents, educational level, age and amount of siblings). There was no relation between attachment classifications and sociodemographic variables; the effect size measures were low in all cases. Results of the two-sided Fisher’s exact test did not show any significant associations between the two-group attachment classifications (secure-insecure) and gender, educational level, marital status of parents and household. Furthermore, findings on age [*t* test: *t*(77) = 0.461, *p* = .646, η^2^ = .0028, *d* = 0.11] and amount of siblings (exact Mann–Whitney test: *p* = .639, η^2^ = .0024, *d* = 0.10) did not demonstrate a significant relation to secure-insecure attachment classifications (Table 1).

**Table 1:** Two-sided Fisher’s exact test for securely and insecurely-attached adolescents on the sociodemographic variables gender, household, marital status of parents and educational level

Variables	Secure^a^	Insecure^b^	Φ	*p*
*Gender*				
Male	10	11	.07	.61
Female	23	35		
*Household*				
Living with their parents	29	38	.07	.75
Single/shared apartment	4	8		
*Marital status of parents*				
Married parents	26	35	.03	1.000
Not married or single parents	7	11		
*Educational level*				
Education with matura	29	43	.10	.44
Education without matura	4	3		

*Φ* = Cramer’s V, *p* = significance, *Matura* general qualification for university entrance in Austria

* *p* < .05; ** *p* < .01

^a^N = 33; ^b^ N = 46

For the four-group classification (F, Ds, E, U), the results also did not demonstrate any significant relation to (a) gender (*p* = .831, Φ = .11) (b) age [*F*(3,75) = 0.625, *p* = .601, η^2^ = .024], (c) educational level (*p* = .738, Φ = .14), (d) marital status of parents (*p* = .556, Φ = .16), (e) number of siblings (*p* = .963, η^2^ = .0047) and (f) household (*p* = .109, Φ = .14).

### Verbal Intelligence, Social Desirability and Verbal Fluency

Our core interest in this study focused on evaluating associations between AAP classification and verbal intelligence (verbal comprehension index, VCI, *M* = 114.8, *SD* = 10.0), social desirability (BIDR, *M* = 76.5, *SD* = 13.0), and verbal fluency operationalized as a total story length in words (*M* = 1137.0, *SD* = 428.3, range 516–2633 words). The exact two-sided Kolmogorov–Smirnov test indicated no violations of the distribution normality by VCI (*p* = .883) and by BIDR (*p* = .761). Story length was significant (*p* = .043); however, no significant violation was indicated following logarithmic transformation (*p* = .578). Hence, we operationalized the verbal fluency as logarithm (base 10) of story length in words (*M* = 3.030, *SD* = 0.147). Before testing the hypothesis, we calculated possible correlations between verbal intelligence, social desirability and verbal fluency. Verbal intelligence was not significantly correlated with verbal fluency [*r*(77) = −0.038, *p* = .737] or social desirability [*r*(77) = .020, *p* = .862]. However, there was a significant correlation between social desirability and verbal fluency [r(77) = 0.238, *p* = .035]. We next examined the effects of gender and age on verbal intelligence, social desirability and verbal fluency. Gender and age were not significantly related to social desirability or verbal fluency. However, there was a significant negative correlation between age and verbal intelligence [*r*(77) = −.410, *p* < .001].

We ran a series of ANCOVA to examine the relation between AAP classifications, both 4- (F, Ds, E, U) and 2-group classifications (secure-insecure), and verbal intelligence, social desirability and verbal fluency. Attachment classification was considered as the grouping factor for each dependent variable (VCI, BIDR, verbal fluency) and gender and age (in years) were considered as covariates. Means and standard deviations for the VCI, BIDR and verbal fluency for the four- (F, Ds, E, U) and 2-group classifications (secure-insecure) are presented in Tables 2 and 3.

**Table 2:** Means and standard deviations of verbal intelligence, social desirability and verbal fluency for the four group classifications (F, Ds, E, U)

	F		Ds		E		U		F	*df*	*p*
	M	SD	M	SD	M	SD	M	SD			
VCI	114.88	9.50	113.07	10.98	121.20	7.44	112.89	9.37	1.58	3	.20
BIDR	74.52	13.97	80.00	12.27	74.20	10.41	75.89	13.44	1.02	3	.40
VF	1240.61	536.92	1120.81	299.81	886.30	207.26	1084.56	410.06	1.92	3	.13

*F* secure, *Ds* dismissing, *E* preoccupied, *U* unresolved, *VCI* verbal comprehension index, *BIDR* balanced inventory of desirable responding, *VF* verbal fluency

* *p* ≤ .01; ** *p* ≤ .001

^a^N = 33, ^b^ N = 27, ^c^ N = 10, ^d^ N = 9

**Table 3:** Means and standard deviations of verbal intelligence, social desirability and verbal fluency for the two group classifications (secure-insecure)

	Secure^a^		Insecure^b^		F	*df*	*p*
	M	SD	M	SD			
VCI	114.88	9.50	114.84	10.40	.01	1	.91
BIDR	74.52	13.97	76.51	12.14	1.51	1	.22
VF	1240.61	536.92	1137.04	428.30	2.81	1	.10

*VCI* verbal comprehension index, *BIDR* balanced inventory of desirable responding, *VF* verbal fluency

* *p* ≤ .01; ** *p* ≤ .001

^a^N = 33, ^b^ N = 46

There were neither significant differences on the VCI among the four attachment groups (*F*(3, 73) = 1.581, *p* = .201, η_p_^2^ = .061, 90 %-CI [.000, .136]) nor between the two attachment groups (*F*(1, 75) = .012, *p* = .913, η_p_^2^ = .00016, 90 %-CI [.000, .032]). Furthermore, we did not find any significant difference of BIDR scores among the four attachment groups (*F*(3, 73) = 1.019, *p* = .389, η_p_^2^ = .0040, 90 %-CI [.000, .102]) and among the two attachment groups (*F*(1, 75) = 1.514, *p* = .222, η_p_^2^ = .020, 90 %-CI [.000, .097]).

We alternatively hypothesized that secure adolescents might present stories in a more coherent way because of more advanced verbal fluency, logical thinking and verbal expression. However, no significant differences among the four attachment groups on verbal fluency (*F*(3, 73) = 1.923, *p* = .133, η_p_^2^ = .073, 90 %-CI [.000, .153]) were found. Yet interestingly, there was a trend for secure adolescents to demonstrate greater verbal fluency than insecure adolescents, however this finding did not reach statistical significance (*F*(1, 75) = 2.809, *p* = .098, η_p_^2^ = .036, 90 %-CI [.000, .126]).

## Discussion

The study of attachment-related aspects of adolescent development, psychopathology, and intervention has received increasing attention in recent years. Researchers have already used the AAP in adolescents as it is economical in its use and it provides valuable information on states of mind regarding attachment that can be applied in the clinical context. For the first time, this preliminary study examined the discriminant validity of the AAP in adolescents to make this instrument feasible for younger populations. This validation study followed a study design analogous to the design used to validate the AAI [42, 45], the established “gold standard” measurement for adult attachment and the predominant measure used in adolescent attachment research.

One aim of this study was to investigate distribution of attachment classifications in a non-risk adolescent sample. To compare it to adult data, we used adolescent German versions of instruments that were also used in the North American AAP adult validation study [31]. The distribution of attachment classification patterns in our sample participants was analogous to the distributions reported in studies of non-clinical adolescents that measured attachment using the AAI [28]. It is important to emphasize that we do not interpret this close similarity of both distributions, which were collected in different samples, in the sense of the convergent validity. Nevertheless is interesting that we found a similar distribution although a different distribution would not challenge the validity of the AAP. One future direction regarding further establishing the validity of the AAP is to investigate correlations between AAI and AAP classifications in adolescence. Although data from adult studies already demonstrated acceptable concurrent validity [31], this has not been published for a younger age (<18 years)group yet. Our study group is currently conducting a study on classification concordance rates between AAI and AAP in an adolescent sample.

The results from our validity testing demonstrated acceptable discriminant validity for adolescents and contribute significantly to the findings on the psychometric properties of the AAP in adults [11, 31, 33, 36, 53]. Attachment classification using the AAP relies on evaluations of discourse responses to the stimuli and discourse production that may be influenced by verbal intelligence. Previous studies have already demonstrated that verbal intelligence is not related to response production in adults, neither in the AAP nor in the AAI [31, 42]. Therefore, establishing discriminant validity testing verbal intelligence was a first important step in validating the AAP for adolescents. As mentioned before, verbal intelligence might pose challenges especially for adolescents, since studies showed fluctuations during teenage years. It was alternatively hypothesized that adolescents with high VCI scores might be more likely to be judged secure than insecure by virtue of being able to construct logically consistent stories and integrated descriptions of story themes and characters. Our results did not demonstrate, however, any significant differences on any of the VCI dimensions among the four attachment groups. We also evaluated the question of whether the narrative length of the story (i.e., number of words produced) was related to attachment classification. The narrative style of protocols can differ considerably inter-individually and a good storyteller might present very imaginative and verbally complex stories. Thus, it was alternatively hypothesized that longer and richer stories might be related to secure attachment, whereas shorter and less descriptive stories might be associated with insecure attachment. Our results demonstrate no significant correlation between story length and attachment classifications, supporting the hypothesis that fluency, vocabulary breadth, and narrative elaboration is not associated with attachment classification in adolescents.

Adolescence has been described in research as a time during the life span punctuated by the motivation for social acceptance and presenting the self in socially desirable ways [44]. We examined this developmental phenomenon by analyzing social desirability using the BIDR to test the alternative hypothesis that AAP classifications are influenced by adolescents’ tendencies to present the self in a socially desirable light. Our results showed, as expected, no significant differences among the attachment groups on social desirability. In other words, the degree to which adolescents deny socially undesirable traits or portray themselves in a favorable light is not associated with attachment classification groups on the AAP. We also tested possible interactions between social desirability and verbal intelligence, as adolescents who tend to give socially desirable responses in the AAP might also have a higher VCI and thus produce more coherent and less inconsistent story lines in response to the picture stimuli that is usually attributed to a secure attachment pattern. We found no significant correlations between BIDR and VCI scores. This result suggests that adolescents who tend to answer in a socially desirable way do not necessarily produce more coherent stories that might skew classification in favor of security.

Certain limitations must be taken into account in interpreting the findings of this study. First, the transcription and the following detailed expert rating of verbatim text protocols demands considerable time resources; therefore, the sample size of our study is– compared to questionnaire methods—with 79 cases relatively small for a validity study. There is a possibility that non-significant results supportive of discriminant validity are a function of sample size. For this reason, we included the confidence intervals for the eta-square coefficients both for secure-insecure and four-group AAP classifications showing exactly the possible extent of the influence of the sample size on the principal study results. The interval estimations were sufficiently precise; nevertheless, the size of the sample and attachment representation subgroups limited the application of simultaneous methods. Moreover, the sample consisted of low-risk mainly middle-class subjects with relatively limited ethnic diversity and an overrepresentation of girls. Whereas sociodemographic variables like gender, age, or educational level were not significantly associated with attachment classification, influences of cultural diversity and psychological risk need to be examined in future studies. As our sample consisted of more girls than boys, we adjusted the effects of gender in our analyses by using gender as covariates. ANCOVA analyses did not show any significant effect of gender on the relationship between AAP classifications, verbal intelligence, and social desirability. This result is consistent with a large number of AAI studies that also show no influence of gender on attachment classifications in adults [28]. Future studies should include young people from lower socioeconomic samples, from different ethnic backgrounds, and clinical groups to examine validity in samples with a greater range of characteristics on sociodemographic variables that we presented in the current study. Our sample was recruited by distributing flyers in front of schools and sending emails via a mailing list. It is possible that our recruitment procedure was the source of our homogenous sample. Futures research should consider expanding the recruitment to adolescent venues and groups that are not defined by education and email accessibility, including teens at higher sociodemographic and developmental risk, than those that participated in the current study.

Second, our sample consisted of German-speaking adolescents and it must be considered that the majority of AAP studies were done with adults in North American samples (English speaking and French speaking participants). However, one study conducted in Germany examined the neural correlates of resolved versus unresolved attachment on 34 cases classified by a German-speaking and by an English-speaking reliable judge [34]. This study reported a 91 % statistically significant classification concordance rate of 91 % (κ = .81, *p* < .001) [34]. This finding suggests that a German cultural background does not necessarily influence attachment classifications in the AAP in adults. In addition, there seems to be an indirect evidence supporting the finding of cultural validity of the AAP from a study examining classification concordance with German language AAI interviews [31]. The AAI rating was based on German transcripts. Results demonstrate a statistically significant correspondence rate between AAP and AAI of 84 % for the four-group classification (κ = .71, *p* < .001), 91 % for secure versus insecure (κ = .70, *p* < .001) and 88 % for unresolved versus resolved (κ = .75, *p* < .001) [34]. These results fortify Bowlby’s [38, 63] proposition that attachment security can be assessed using similar measures cross-culturally as these instruments function similarly among different cultures. Nevertheless, research has demonstrated that there are cross-cultural variations in attachment–related parenting behaviour, although no cultural differences in representational attachment assessment have been demonstrated to date [23]. So far, no study has conducted AAP interviews in other cultural regions and especially in an adolescent age group and thus represents an important research field regarding further establishing the validity of the AAP.

Third, this study did not examine AAP test–retest reliability. Although test–retest reliability was tested for the AAP in adults [31], this question must be examined for adolescents as well. Similarly, predictive validity data of the AAP is available for adults (for an overview see George and West [39]), and for the first time it is tested in an ongoing longitudinal study on transgenerational transmission of maltreatment in 200 mothers and their children [64], more work is required to provide predictive validity evidence of the AAP for adolescence. Given the changes that occur in parental and peer relationships during adolescence, studies that focus on fundamental aspects in that age group such as parent-youth interactions or the degree of social competence with peers might be a promising direction for future research [17].

Today, the role of attachment, its assessment, and clinical applications for adolescent populations are still relatively untapped areas of research [9]. In the current literature, there has been substantially more research using self-report measures of attachment in adolescents, which are often criticized for problems with subjectivity and difficulty in integrating empirical findings to developmental attachment constructs [1]. At the same time, a growing number of narrative-based papers provide strong evidence of a high prevalence of the unresolved attachment pattern not only in adolescents with mental disorders but also in their parents [2, 8, 65]. The nature of these traumatizing events related to attachment and the role of the unresolved classification- that can only be assessed using a narrative instrument- is still poorly understood and represents a promising direction for future adolescent attachment research especially in clinical settings. Using narrative techniques provides a deeper understanding of unconscious aspects of attachment-related defenses and unresolved attachment status that might lay out the foundation for developing attachment-based therapeutic interventions for adolescents. Although there are well-established narrative interview techniques (e.g., AAI, CAI), they are time-consuming and costly [52]. Therefore, we suggest that the results of this preliminary validation study poises the AAP as a viable tool for basic and clinical research with adolescence; it is user-friendly, economical, and has demonstrated impressive agreement with the AAI in adults [31]. Furthermore, studies such as this one that establishes preliminary psychometric validity for using the AAP with adolescents add increased credibility to predictive validity and clinical adolescent studies [7, 10, 11].

Future research employing the AAP for assessing attachment would make important contributions to our understanding of adolescent development, especially psychopathology risk, and lay out the foundation for developing attachment-based therapeutic practices for this age group. Following this idea some studies have included the AAP into assessment and treatment in clinical settings [66–69]. A growing body of research have demonstrated the utility of the AAP to design specialized intervention plans, predict compliance and illustrated how a deeper understanding of attachment issues help the clinician predicting therapeutic alliance and thinking about treatment options [7, 10]. Following these results some studies have included the AAP into the assessment and treatment in clinical settings [66–69]. As the AAP provides an insight into an individual’s traumatic dysregulation and defensive structures, therapeutic interventions could focus step by step on supporting patients to understand their emotional reactions of helplessness in the context of their treatment setting.

Although some of studies offer preliminary data on the use of the AAP in adolescents [7, 10, 11], more research is needed on the role of attachment for psychopathology, its assessment and clinical application in that age group [9, 70]. Our findings demonstrate an acceptable discriminant validity of the AAP for assessing adolescent attachment representation with no associations between attachment classification and verbal intelligence, story length, social desirability, and sociodemographic variables, making this instrument feasible for use in a wide range of clinical settings including younger patients. Furthermore, we found a good inter-rater reliability for our adolescent population. Future research employing this narrative instrument for assessing attachment would make important contributions to our understanding of adolescent psychopathology and lay out the foundation for developing attachment-based therapeutic practices for teenagers.

## Summary

The contribution of attachment to human development and clinical risk is well established for children and adults, yet there is relatively limited knowledge about attachment in adolescence. A major contributor to this phenomenon is poor availability of construct valid attachment measures for this age group. The AAP is a validated free-response measure of attachment status based on “story” responses to a battery of seven picture stimuli depicting attachment situations [31, 33]. The current study is the first study to examine the discriminant validity using the AAP with adolescents. First, we calculated the distribution of attachment. In our sample of 79 adolescents between 15 and 18 years, 42 % were classified as secure, 34 % as insecure-dismissing, 13 % as insecure-preoccupied and 11 % as unresolved. This distribution is analogous to the distribution reported in studies of non-clinical adolescents that measured attachment using the AAI. Second, we analyzed possible relations between attachment classification and verbal intelligence, social desirability, story length and sociodemographic data. The narrative style of AAP protocols—verbatim transcripts of stories associated to the stimulus pictures—can differ considerably inter-individually. A “good adolescent storyteller” can present stories with an imaginative content, stories that are verbally complex and moreover intentionally attractive for the listener. It would be natural to suspect that such “good adolescent storytellers” can reach the secure classification in AAP more easily. Our study findings demonstrate that attachment classification is associated neither with verbal intelligence and productivity nor with social desirability and sociodemographic variables. These results poise the AAP to be used in clinical intervention and large-scale research investigating normative and atypical developmental correlates and sequelae of attachment, including psychopathology in adolescence. Future research employing this narrative instrument for assessing attachment would make important contributions to our understanding of adolescent psychopathology and lay out the foundation for developing attachment-based therapeutic practices for teenagers
